# Aerobic Composting and Anaerobic Digestion Decrease the Copy Numbers of Antibiotic-Resistant Genes and the Levels of Lactose-Degrading Enterobacteriaceae in Dairy Farms in Hokkaido, Japan

**DOI:** 10.3389/fmicb.2021.737420

**Published:** 2021-09-30

**Authors:** Satoshi Katada, Akira Fukuda, Chie Nakajima, Yasuhiko Suzuki, Takashi Azuma, Ayaka Takei, Hideshige Takada, Eiryu Okamoto, Toshihide Kato, Yutaka Tamura, Masaru Usui

**Affiliations:** ^1^Laboratory of Food Microbiology and Food Safety, School of Veterinary Medicine, Rakuno Gakuen University, Ebetsu, Japan; ^2^Division of Bioresources, Hokkaido University Research Center for Zoonosis Control, Sapporo, Japan; ^3^International Collaboration Unit, Research Center for Zoonosis Control, Hokkaido University, Sapporo, Japan; ^4^Department of Pharmacy, Osaka Medical and Pharmaceutical University, Takatsuki, Japan; ^5^Laboratory of Organic Geochemistry, Faculty of Agriculture, Tokyo University of Agriculture and Technology, Fuchu, Japan; ^6^Laboratory of Environmental Microbiology, College of Agriculture, Food, and Environment Sciences, Rakuno Gakuen University, Ebetsu, Japan; ^7^Department of Large Animal Clinical Sciences, School of Veterinary Medicine, Rakuno Gakuen University, Ebetsu, Japan

**Keywords:** aerobic composting, ampicillin resistance, anaerobic digestion, antimicrobial resistance, dairy cow, tetracycline resistance

## Abstract

Efficient methods for decreasing the spread of antimicrobial resistance genes (ARGs) and transfer of antimicrobial-resistant bacteria (ARB) from livestock manure to humans are urgently needed. Aerobic composting (AC) or anaerobic digestion (AD) are widely used for manure treatment in Japanese dairy farms. To clarify the effects of AC and AD on antimicrobial resistance, the abundances of antimicrobial (tetracycline and cefazolin)-resistant lactose-degrading Enterobacteriaceae as indicator bacteria, copy numbers of ARGs (tetracycline resistance genes and β-lactamase coding genes), and concentrations of residual antimicrobials in dairy cow manure were determined before and after treatment. The concentration of tetracycline/cefazolin-resistant lactose-degrading Enterobacteriaceae was decreased over 1,000-fold by both AC and AD. ARGs such as *tetA*, *tetB*, and *bla*_*TEM*_ were frequently detected and their copy numbers were significantly reduced by ∼1,000-fold by AD but not by AC. However, several ARG copies remained even after AD treatment. Although concentrations of the majority of residual antimicrobials were decreased by both AC and AD, oxytetracycline level was not decreased after treatment in most cases. In addition, 16S rRNA gene amplicon-based metagenomic analysis revealed that both treatments changed the bacterial community structure. These results suggest that both AC and AD could suppress the transmission of ARB, and AD could reduce ARG copy numbers in dairy cow manure.

## Introduction

Antimicrobials are used for treating bacterial infections and promoting healthy development in livestock animals. According to a recent report, a total of 825 tons of antimicrobials have been used to treat livestock animals in 2018 in Japan (Ministry of Health, Labour and Welfare, 2020). Excessive use of antimicrobials in livestock has led to increased circulation of antimicrobial-resistant bacteria (ARB) and antimicrobial resistance genes (ARGs) (Woolhouse et al., 2015). Most consumed antimicrobials pass through the animal unmetabolized and are excreted in manure (Congilosi and Aga, 2020). Livestock manure is one of the main sources of ARB, ARGs, and antimicrobials released into the environment (Zhao et al., 2010; Congilosi and Aga, 2020). Infection of humans by ARB from livestock and subsequent transfer of ARGs to human microbiota have been reported and is an important public health concern (Woolhouse and Ward, 2013).

Livestock manure is generally treated by aerobic composting (AC) and anaerobic digestion (AD). After treatment, the compost is usually used for soil fertilization. However, the treated compost contains ARB and ARGs, and it could contribute to their spread in agricultural land (Hou et al., 2015). In addition, residual antimicrobials in the soil could trigger the emergence and proliferation of ARB, as well as supporting the persistence and increase of ARG copy numbers, in the environment (Congilosi and Aga, 2020). ARB and ARGs in the soil can contaminate agricultural products and can be transmitted to humans through the food chain (Yang et al., 2016; Zhang Y. J. et al., 2019). Furthermore, wild animals and insects close to the farms could become reservoirs of ARB and ARGs and, thereby contaminate the neighboring environments (Rogers et al., 2018). Therefore, it is important to reduce the abundances of ARB, ARGs, and residual antimicrobials before applying treated composts to the soil.

When livestock manures are subjected to AC, which is widely implemented in Japan, the temperature of the manure is raised by self-heating, resulting in the removal of most of the pathogens (Congilosi and Aga, 2020). It is well known as a suitable method of stabilizing livestock manure substrates to produce nutrient-rich fertilizer. In AD, livestock manures are treated at a constant temperature. AD has an advantage in that it can provide energy in the form of methane gas, but it is more expensive than AC because of the costs associated with the construction and maintenance of the biogas plant (Congilosi and Aga, 2020). The digested manure is a valuable fertilizer that is often spread in agricultural lands to improve soil quality and introduce nutrients for the crops. In Japan, AD has been popularized, especially in suburban areas, and since 2000, its use has been supported by environmental policies.

We have demonstrated previously that ARB content in pig feces was decreased by AC (Yoshizawa et al., 2020). Other reports have also shown that AC and AD impact the abundance of ARB, ARGs, and residual antimicrobials in livestock manure (Congilosi and Aga, 2020). Several studies have demonstrated that AD significantly reduce ARG copy numbers in animal manure (Diehl and LaPara, 2010; Ma et al., 2011). However, other reports have documented increased abundance of ARGs in AD-treated manure (Aydin et al., 2015; Cheng et al., 2016; Sui et al., 2016). The effects of treatments vary depending on various factors (Sharma et al., 2009; Ma et al., 2011). For example, AC in Japan is frequently combined with rotation, which is not the case in other countries. Therefore, to clarify the effectiveness of AC and AD against ARB, ARGs, and residual antimicrobials, collecting and analyzing relevant data from different areas is important.

Although most of the current reports about livestock manure treatments are from chicken or pig farms, dairy cow manure is also an important reservoir of ARB and ARGs (Wichmann et al., 2014; Chambers et al., 2015). Dairy cow manure accounts for about 30% of total livestock manure in Japan. It is therefore important to assess the efficiency of manure treatment practices in removing antimicrobial resistance factors (ARB, ARGs, and residual antimicrobials) from cow manure prior to its application in fields. Therefore, we analyzed the current information about livestock manure treatments in dairy farms in Japan.

In this study, the abundance of ARB, ARGs that confer resistance to tetracycline (TC) or cefazolin (CEZ), and residual antimicrobials were determined in manure samples before and after AC and AD. TC and CEZ were selected because these antimicrobials are frequently used in dairy farms (Ministry of Agriculture, Forestry and Fisheries, 2019). TC is the most widespread veterinary antimicrobial in Japan, and CEZ is commonly used for mastitis treatment. In addition, 16S rRNA gene amplicon-based metagenomic analysis was performed to investigate the change in microbiota induced by AC and AD.

## Materials and Methods

### Sample Collection

In general, all samples were collected four times, i.e., in spring (May), summer (August), autumn (November), and winter (February), between May 2019 and February 2020 at four dairy cow farms in Hokkaido, Japan as Hokkaido prefecture has the most active dairy farms in Japan. Information about the tested samples is provided in Supplementary Table 1. In these farms, livestock manure is subjected to AC (all four farms) and AD (three farms; farm A, B, and D). In all four farms, manure was collected at the early stage of AC (pre-composting) and after composting for around 40 days (post-composting). Before sampling, each sample was mixed well from the surface to the center. Next, approximately 50 g of samples were collected in sterilized tubes. In the three farms that implemented AD, approximately 50 g of livestock manure was collected in sterilized tubes from the plants before AD, and the samples were separated into liquid and solid components (pre-AD of liquid/solid). After anaerobic digestion for 25–40 days at a mesophilic temperature (35–40°C), approximately 50 mL of the liquid components of livestock manure were collected from the storage tank (post-AD of liquid). In two out of three AD performing farms, treated manure was also separated into liquid and solid components. Approximately 50 g of the solid component (post-AD of solid) was collected in sterilized tubes from the piles. However, because they were obtained from filed samples, the temperature changes and conditions during the composting and anaerobic digestion processes were not determined. All collected samples were examined for bacterial tests and DNA extraction on the day of collection. All samples were stored at −80°C until they were chemically analyzed.

### Bacterial Abundance and Antimicrobial-Resistant Bacteria Abundance

To determine the abundance of lactose-degrading Enterobacteriaceae including *Escherichia coli*, 0.2 g of each sample was suspended in 1 mL of sterile saline. Suspensions were serially diluted and plated on three different types of deoxycholate hydrogen sulfide-lactose (DHL) agar (Nissui Pharmaceutical Co., Ltd., Tokyo, Japan) with or without antimicrobials [16 μg/mL TC (Sigma-Aldrich, MO, United States) or 8 μg/mL CEZ (Sigma-Aldrich)] to estimate the counts (CFU/g) of total lactose-degrading Enterobacteriaceae, TC-resistant lactose-degrading Enterobacteriaceae, and CEZ-resistant lactose-degrading Enterobacteriaceae. The concentrations of added antimicrobials were decided according to the Clinical and Laboratory Standards Institute breakpoints for estimating the levels of antimicrobial-resistant *E*. *coli* (Clinical Laboratory Standards Institute [CLSI], 2020). The colonies were counted and identified as lactose-degrading Enterobacteriaceae according to the colony morphology (red colonies) after incubating for 20 h at 37°C. In addition, bacterial identification for some randomly picked colonies was conducted using matrix-assisted laser desorption/ionization-time of flight mass spectrometry with a Bruker MALDI Biotyper system (Bruker Daltonics, Bremen, Germany) according to the manufacturer’s instructions.

### DNA Extraction

DNA was extracted using ISOFECAL for Beads Beating (Nippon Gene Co., Ltd., Tokyo, Japan) according to the manufacturer’s instruction with some modifications. Briefly, DNA was extracted from 0.2 g compost/solid samples or 200 μL liquid component samples. Lysing Matrix B (MP BioMedicals, Irvine, CA, United States) was used instead of Beads Tube in the kit to increase DNA concentration because it can lyse bacteria more efficiently. Extracted DNA samples were stored at −20°C until use.

### qPCR Analysis

Copy numbers of two TC resistance genes (*tetA* and *tetB*), three β-lactam resistance genes (*bla*_*TEM*_, *bla*_*SHV*_, and *bla*_*CTX-M*_) were quantified by qPCR. These genes were selected as they are commonly associated with tetracycline resistance and β-lactam resistance in Gram-negative bacteria and frequently isolated from livestock. The standard curves were generated by cloning amplicon from the genes (amplified from the laboratory strains in Supplementary Table 2 using the primers detailed in Supplementary Table 3) into the pTA2 vector and transforming them into *E*. *coli* DH5 competent cells using a Target Clone kit (Toyobo, Osaka, Japan). Transformants harboring the recombinant plasmid were selected, and the plasmids were extracted by using a QIAGEN Plasmid Midi Kit (Qiagen, Hilden, Germany) according to the manufacturer’s instruction. The concentration of the purified recombinant plasmid DNA was determined using a Qubit 4 Fluorometer (Thermo Fisher Scientific K.K., Tokyo, Japan), and the plasmids were then used as qPCR standards. Next, qPCR was performed using TB Green Premix Ex Taq II (Tli RNaseH Plus; TaKaRa, Shiga, Japan) in 20 μL of reaction mixtures containing 5 μL of DNA template and 0.4 μM of each primer (Supplementary Table 3). Analyses were performed using a Light Cycler 480II instrument and its software (Roche-Diagnostics, Basel, Switzerland). Reaction conditions were as follows: initial denaturation at 95°C (30 s) was followed by annealing for 45 cycles of 95°C (5 s each) and elongation at appropriate temperatures (Supplementary Table 3) for 30 s. The melting curves were generated and analyzed to verify that no non-specific amplification had occurred. The amplification efficiency, *R*^2^ value, and limit of quantification of each tested gene are provided in Supplementary Table 4. Analyses were performed three times in duplicate and the mean and standard errors were calculated.

### Determination of Concentrations of Residual Antimicrobials

Aerobic compost and solid component samples (0.1 g) were mixed with 5 mL of distilled water. Ultrasonic extraction from the mixtures was performed using a UT-206H ultrasonic washing machine (Sharp Corporation, Osaka, Japan) at 40°C for 10 min. The extract was filtered using a Whatman GD/X filter (Sigma-Aldrich). The samples were pre-treated with Oasis HLB sorbent (Waters Corporation), and then the concentrations of TCs (chlortetracycline, doxycycline, minocycline, oxytetracycline, and tetracycline) and β-lactams (ampicillin, benzylpenicillin, cefazolin, cefuroxime, and ceftiofur) in the extract were determined using ultra-performance liquid chromatography tandem mass spectrometry (UPLC-MS/MS) (Waters Corporation).

The liquid component samples were centrifuged at 2,500 rpm for 10 min and filtered under negative pressure through a Whatman GF/F filter (Sigma-Aldrich). Samples were pre-treated with Oasis HLB sorbent (Waters Corporation, MA, United States), and then concentrations of antimicrobials (TCs, quinolones, sulfa drugs, and macrolides) in filtration samples were determined using LC-MS/MS (Thermo Fisher Scientific K.K).

Six-point standard calibration curves were constructed for quantification ranging from 0.5 to 200 ng/mL. Individual linear calibration curves for each compound were obtained in the concentration range from 0.5 to 200 ng/mL (*r*^2^ > 0.99). Quantification was performed by subtracting the blank data from the corresponding data obtained from the spiked sample solutions to account for matrix effects and losses during sample extraction (Petrović et al., 2014; Azuma et al., 2019). Similarly, rates of recovery were calculated from the deviations between data from the spiked samples and standard solution to perform the calibration. Recovery rates ranged from 59 to 102%; these profiles were generally similar to those reported in previous studies of antimicrobials in compost samples (Pan et al., 2011; Gros et al., 2013; Zhang M. et al., 2019). The limits of detection (LOD) and limits of quantification (LOQ) were calculated as the concentrations at signal-to-noise ratios of 3 and 10, respectively (de Jesus Gaffney et al., 2015; Schlüsener et al., 2015). These values are also summarized in Supplementary Table 5.

### Bacterial Community Analysis

High-throughput sequencing of bacterial communities was performed by Miseq (Illumina, Inc, MA, United States). The following steps were performed according to the 16S Metagenomic Sequencing Library Preparation kit (Illumina, Inc.). The V3–V4 regions of 16S rRNA gene were amplified by amplicon PCR using the 2 × KAPA HiFi HotStart Ready Mix (Kapa BioSystems, Inc, MA, United States) with the forward primer: 5′-TCGTCGGCAGCGTCAGATGTGTATAAGAGACAGCCTA CGGGNGGCWGCAG-3′ and reverse primer: 5′-GTCTC GTGGGCTCGGAGATGTGTATAAGAGACAGGACTACHVGG GTATCTAATCC-3′. The products were cleaned up by AMPure XP beads (Beckman Coulter Inc, CA, United States). Then, dual-index barcodes and Illumina sequencing adapters were added by index PCR, and the products were cleaned up by AMPure XP beads. All samples were pooled as a library and denatured. Then, the samples were loaded onto Miseq (Illumina, Inc) and runs were performed. Sequence numbers between samples were normalized. Then, phylogenetic analysis as well as α and β diversity analysis were performed by QIIME 2 (Bolyen et al., 2019).

### Statistical Analysis

All statistical analyses in this study were conducted with the significance level fixed at α = 0.05 (*P* < 0.05). The Wilcoxon signed-rank test was used to determine the significance of differences in bacterial counts, ARG copy numbers, and the relative abundance of bacteria at the order level before and after treatment. Bacterial counts and ARG copy numbers before treatment were divided by the corresponding values after treatment, and the ratios were used in the Wilcoxon signed-rank test to evaluate the significance of the differences between the seasons in which the samples were collected. The Pearson test was used to analyze correlations between the copy numbers of ARGs and concentrations of antimicrobials. Based on the weighted Unifrac distances from next-generation sequencing, principal coordinate analysis (PCoA) and PERMANOVA were performed to assess the significance of differences in bacterial communities before and after treatment for each sample type. Furthermore, the Mantel test was used to analyze correlations between weighted UniFrac distance and Bray-Curtis dissimilarity calculated from the copy numbers of *tetA*, *tetB*, and *bla*_*TEM*_. The network analysis was performed based on Spearman coefficients of correlation between ARG copy number and relative abundance of dominant (detected in >50% samples) bacteria at the order level. QIIME 2 was used for PCoA and PERMANOVA, and R software was used for other analyses.

### Biosecurity and Institutional Safety Procedure

In this study, standard biosecurity and institutional safety measures were taken in all experiments.

## Results

### Bacterial Abundance

**FIGURE 1:**
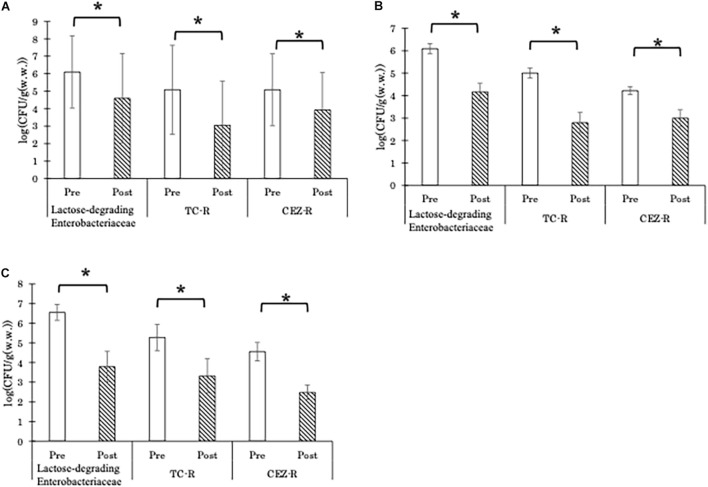
Abundance (CFU/g) of total lactose-degrading Enterobacteriaceae, tetracycline-resistant lactose-degrading Enterobacteriaceae (TC-R), and cefazolin-resistant lactose-degrading Enterobacteriaceae (CEZ-R) in the aerobic compost group [**A**; *n* = 15 and 16, for pre-treatment (Pre) and 16 for post-treatment (Post), respectively], liquid component in the anaerobic digestion group (**B**; *n* = 12 for both Pre and Post), and solid component in the anaerobic digestion group (**C**; *n* = 8 and 6, for Pre and Post, respectively). Data are presented as the mean ± standard error of the mean. Asterisks (*) indicate significant differences in the abundance before and after treatment.

Both AC and AD significantly decreased the CFU counts of total lactose-degrading Enterobacteriaceae, TC-resistant lactose-degrading Enterobacteriaceae, and CEZ-resistant lactose-degrading Enterobacteriaceae (Figure 1). For both types of treatment, the differences in the reduction of lactose-degrading Enterobacteriaceae abundances between seasons were not significant (Supplementary Figure 1). Of 950 randomly picked red colonies (which are lactose-degrading Enterobacteriaceae), 65.7% were identified as *E*. *coli*.

### Copy Numbers of Antimicrobial Resistance Genes

**FIGURE 2:**
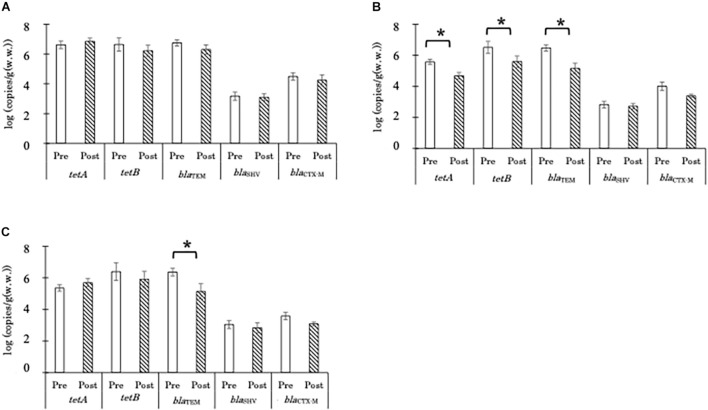
Absolute concentrations of antimicrobial resistance gene copies in the aerobic compost group [**A**; *n* = 15 and 16, for pre-treatment (Pre) and 16 for post-treatment (Post), respectively], liquid component in the anaerobic digestion group (**B**; *n* = 12 for both Pre and Post), and solid component in the anaerobic digestion group (**C**; *n* = 8 and 6, for Pre and Post, respectively). Data are presented as the mean ± standard error of the mean. Asterisks (*) indicate significant differences in the abundance before and after treatment.

The concentrations of *tetA*, *tetB*, and *bla*_*TEM*_ gene copies in aerobic compost were 6–7 log copies/g before and after AC (Figure 2A). AD significantly decreased the copy numbers of *tetA*, *tetB*, and *bla*_*TEM*_ genes, in the liquid component from 5.6, 6.5, and 6.5 copies/g to 4.7, 5.6, and 5.2 log copies/g, respectively (Figure 2B). In the solid component, AD significantly decreased only the number of *bla*_*TEM*_ gene copies, from 6.5 to 5.2 log CFU/g (Figure 2C). The copy numbers of *bla*_*SHV*_ and *bla*_*CTX-M*_ in most of the samples were below the limit of quantification; therefore, no significant effects of either AC or AD treatment were observed (Figure 2). For both types of treatment, the seasonal differences in the reduction in ARG copy numbers were not significant (Supplementary Figure 2).

### Relationships Between Bacterial Communities and Antimicrobial Resistance Genes Copy Numbers

**FIGURE 3:**
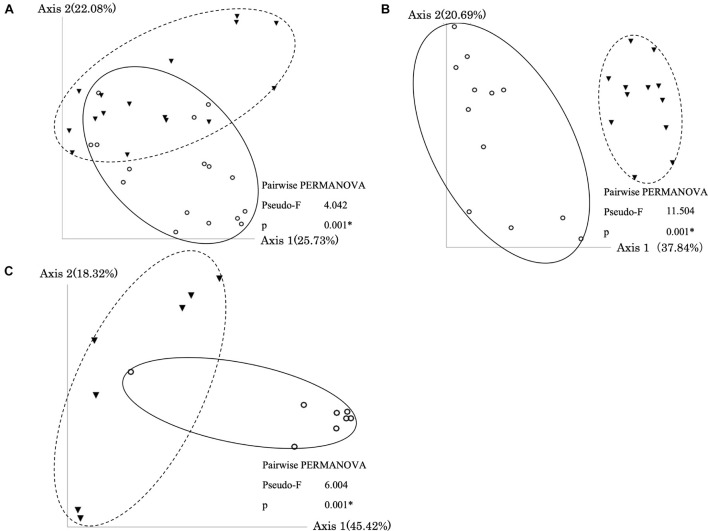
Principal Coordinate Analysis (PCoA) plot and PERMANOVA results based on weighted UniFrac Distance in the aerobic compost group **(A)**, liquid component in the anaerobic digestion group **(B)**, and solid component in the anaerobic digestion group **(C)**. Circles and solid lines indicate samples before treatment. Triangles and dotted lines indicate samples after treatment. Asterisks (*) indicate significant difference in the bacterial communities between samples before and after treatment.

**FIGURE 4:**
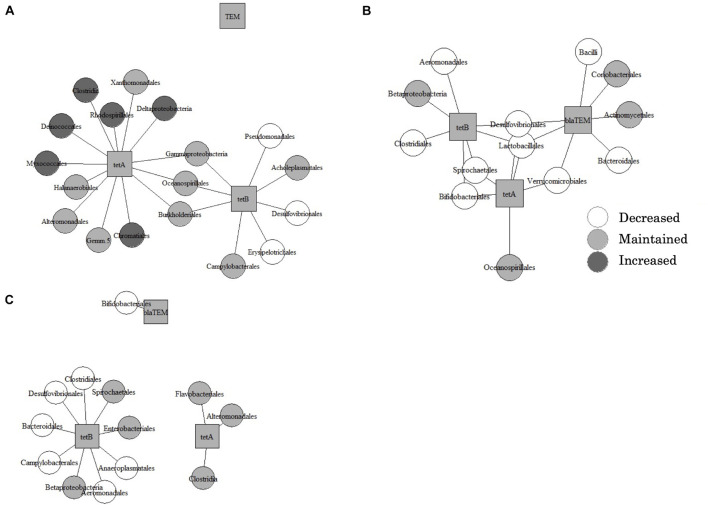
Results of the network analysis in the aerobic compost group **(A)**, liquid component in the anaerobic digestion group **(B)**, and solid component in the anaerobic digestion group **(C)**, showing correlations between bacterial communities and three dominant antimicrobial resistance genes. Bacterial communities are classified as increased, decreased, or unchanged after treatment.

In all types of samples, PCoA and PERMANOVA showed that bacterial communities were significantly changed by the treatment (Figure 3). Furthermore, the Mantel test showed that dissimilarities in bacterial community and dominant ARGs (*tetA*, *tetB*, and *bla*_*TEM*_) were significantly correlated (Supplementary Table 6). The co-occurrence patterns of ARGs and bacterial communities at the order level in three types of samples were investigated using network analysis (Figure 4). Eighteen, thirteen, and thirteen groups showed positive and significant correlation with dominant ARGs (*tetA*, *tetB*, and *bla*_*TEM*_) in AC-treated samples, liquid component subjected to AD, and solid component subjected to AD, respectively (Supplementary Table 7). The correlated communities of three types were observed: those that increased, decreased, and remained without significant changes after treatment. Following AC, of the 18 correlated communities, six bacterial groups (*Rhodospirillales*, *Chromatiales*, *Clostridia*, and others) were increased, nine groups (*Campylobacteriales*, *Burkholderiales*, *Xanthomonadales*, and others) remained the same, and only three groups (*Pseudomonadales*, *Desulfovibrionales*, and *Erysipelotrichales*) were decreased. After AD, no group was increased, 4 out of 13 groups in the liquid component and 6 out of 13 groups in the solid component (including *Actinomycetales*, *Betaproteobacteria*, *Enterobacteriales*, and others) remained stable, whereas other groups (including *Lactobacillales*, *Desulfovibrionales*, and *Bifidobacteriales*) were decreased.

### Residual Antimicrobials and the Effects on Antimicrobial Resistance Genes

**FIGURE 5:**
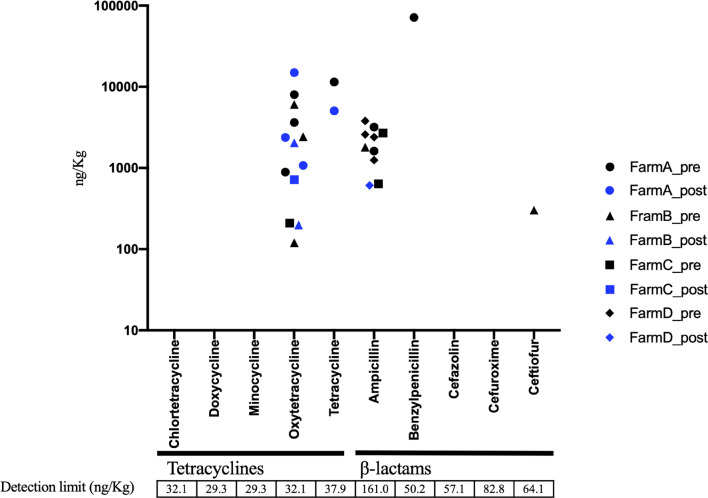
Residual concentration of tetracyclines and β-lactams in aerobic compost (AC) before (*n* = 15) and after treatment (*n* = 16). Black circle, pretreatment in farm A; Blue circle, post-treatment in farm A; Black triangle, pretreatment in farm B; Blue triangle, post-treatment in farm B; Black square, pretreatment in farm C; Blue square, post-treatment in farm C; Black diamond, pretreatment in farm D; Blue diamond, post-treatment in farm D. Unmarked samples are below the detection limit.

**FIGURE 6:**
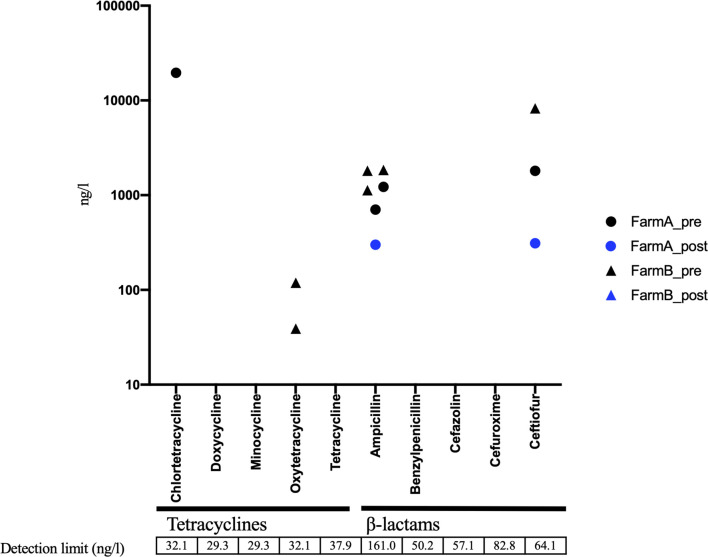
Residual concentration of tetracyclines and β-lactams in solid components subjected to anaerobic digestion (AD) before (*n* = 8) and after treatment (*n* = 6). Black circle, pretreatment in farm A; Blue circle, post-treatment in farm A; Black triangle, pretreatment in farm B; Blue triangle, post-treatment in farm B. Unmarked samples indicate below detection limit.

In AC samples, oxytetracycline was detected in 6 of 15 samples, and ampicillin was detected in 9 out of 15 samples before treatment (Figure 5). The ampicillin level was decreased below the detection limit in eight of the nine positive samples following AC treatment, whereas oxytetracycline remained at the same level in all the six samples (Figure 5). The Pearson test showed positive and significant correlations of the residual level of oxytetracycline with *tetA* and *tetB* levels in aerobic compost samples (Supplementary Figure 3). In the solid component of AD samples, some pre-treatment samples contained detectable amounts of tetracyclines and β-lactams. The concentration of antibiotic (tetracyclines and β-lactams) was decreased to under the detection limit following AD treatment in all samples except 1 sample in which both ampicillin and ceftiofur were decreased (Figure 6). The pre-treatment liquid component samples were not applicable for chemical analysis, because they contained large particles (Supplementary Table 8). In the post-treatment samples, some antimicrobials (minocycline, oxytetracycline, and sulfamethoxazole) were still detected (Supplementary Table 8).

## Discussion

To establish whether dairy cow manure treatment by the methods conventionally adopted in Japan suppresses the spread of antimicrobial resistance, we analyzed the abundance of ARB, ARGs, and antimicrobial drugs in manure samples before and after AC and AD.

The abundance of lactose-degrading Enterobacteriaceae, including antimicrobial-resistant lactose-degrading Enterobacteriaceae, were significantly decreased by both AC and AD. It has been reported that AC completely eliminated *Salmonella* and *E*. *coli* O157:H7 from livestock manure (Edrington et al., 2009), and mesophilic AD removed >90% of multidrug-resistant bacteria (Beneragama et al., 2013). These results suggest that both AC and AD effectively reduce the levels of several bacteria, including *E*. *coli*, in livestock manure. However, lactose-degrading Enterobacteriaceae was not completely eliminated following AC and AD treatments in our study. Therefore, more effective methods to eradicate pathogens such as *E*. *coli* in livestock manure are required.

Copy numbers of three dominant ARGs (*tetA*, *tetB*, and *bla*_*TEM*_) in the liquid component of manure and of *bla*_*TEM*_ in the solid component were significantly decreased by mesophilic AD, but not by AC in this study. These results suggest that AD is superior to AC in reducing the copy numbers of some ARGs. However, all the tested ARGs were still detected in the post-treatment samples, including in those taken after AD. Previous studies have investigated the variations in ARG copy numbers following AC and AD (Diehl and LaPara, 2010). Although AD was reported to decrease the copy numbers of many ARGs (Oliver et al., 2020), mesophilic AD (35–37°C) was found to increase the copy numbers of some ARGs, which were nonetheless decreased by thermophilic AD (55°C) (Sun et al., 2016). AC was shown to have distinct effects on different ARGs; the copy numbers of *tetA* and *tetC* were increased, whereas *tetG* copy number was decreased in dairy cow manure (Sharma et al., 2009). AD yielded a significantly higher reduction in the copy numbers of three TC-associated ARGs compared with AC (Luo et al., 2020). These results suggest that AC and AD does not eliminate ARGs completely. Nevertheless, the hosts of ARGs are not the only bacteria that are affected by AC and AD. The effects of each treatment are likely influenced by the type of ARGs and various environmental factors.

The concentrations of residual ampicillin were decreased by both AC and AD in most of the solid samples, whereas residual oxytetracycline levels were not significantly altered in this study. Furthermore, residual oxytetracycline was also detected after AD in the liquid samples. These results suggest that ampicillin and oxytetracycline were frequently used in the tested farms and these antimicrobials (especially oxytetracycline) remained in livestock waste. This inference is consistent with previous reports that tetracycline residues, but not β-lactam residues, are common and perhaps persistent in dairy cow manure (Oliver et al., 2020). In addition, previous studies reported the variable effects of manure treatment on the persistence of antimicrobials (Congilosi and Aga, 2020). These results suggest that although the levels of antimicrobials in Japanese dairy cow manure are decreased by both AC and AD, some of the antimicrobials, especially tetracyclines, still remain in the treated samples.

Significant relationships between the concentration of residual oxytetracycline and copy numbers of tetracycline resistance genes were observed in this study, which was in agreement with the data from our previous report (Yoshizawa et al., 2020). These results suggest that using antimicrobials in livestock farms exert a selective pressure on ARGs in aerobic composting samples. Moreover, residual antimicrobials in the treated manure may exert a selective pressure on ARG-harboring microorganisms in the soil receiving manure fertilizer (Congilosi and Aga, 2020). A recent study suggested that long-term land deposition of treated digestate increased the relative abundance of ARGs (Congilosi and Aga, 2020). Therefore, it is important to investigate additional manure treatment strategies to improve the removal of not only ARB and ARGs, but also of antimicrobials before using digestate as fertilizer.

Both AC and AD treatments significantly changed the microbial flora. In AC, changes in physicochemical parameters had significant effects on bacterial succession (Meng et al., 2019) and heat-intolerant bacteria (Enterobacteriaceae and Pseudomonas) were removed (Gao et al., 2019). Although the mechanism underlying the change in bacterial flora in AD is complex and unclear, several studies have shown an increase in *Clostridiales* and *Bacteroides* (Sun et al., 2016; Ma et al., 2019). These orders are strictly anaerobic and are involved in the process of methanogenesis. Furthermore, changes in ARG copy numbers and the counts of microbial flora correlated significantly, and it has been suggested that AD affects ARG abundance by changing the microbial population structure (Sun et al., 2016). From the results of network analysis, although the abundance of some groups of potential ARG-containing bacteria (*Lactobacillales*, *Desulfovibrionales*, *Bifidobacteriales*, and others) were reduced by AD, ARGs might be maintained by other bacteria (such as those belonging to *Actinomycetales*) which were stable after AD. Similarly, following AC, some enriched bacteria (*Rhodospirillales*, *Chromatiales*, *Clostridia*, and others) could harbor ARGs and maintain their abundance in the microbiome at the same level.

In conclusion, AC and AD were found to have almost equal capacity for ARB removal from Japanese dairy farm manure. Although it is difficult to eliminate ARB and ARGs from dairy cow manure completely, AD decreased copy numbers of ARGs more strongly than AC. However, it is difficult to encourage the universal use of AD by dairy farmers because of the associated costs. Some reports have suggested modifications of the treatment methods by using specific auxiliary material (Zhang J. et al., 2019), by adding bacteria (Duan et al., 2019) or composting additives (Peng et al., 2018; Bao et al., 2019), or AD (Ma et al., 2019) to reduce antibiotic resistance. Further investigations are needed to find better ways to prevent the spread of antimicrobial resistance in farms.

In summary, both AC and AD could suppress the transmission of ARB, and AD could reduce ARG copy numbers in dairy cow manure by changing the bacterial community structure. However, because ARGs could not be eliminated completely, it is necessary to find highly efficient methods to decrease ARG copy numbers.

## Data Availability Statement

The datasets presented in this study can be found in online repositories. The names of the repository/repositories and accession number(s) can be found below: https://www.ddbj.nig.ac.jp/, DRR308845-DRR308919.

## Author Contributions

SK: investigation and writing. CN and YS: data curation and investigation. AF, TA, AT, and HT: investigation. EO and TK: resources. YT: conceptualization and data curation. MU: conceptualization, data curation, investigation, project administration, resources, and writing. All authors contributed to the article and approved the submitted version.

## Conflict of Interest

The authors declare that the research was conducted in the absence of any commercial or financial relationships that could be construed as a potential conflict of interest.

## Publisher’s Note

All claims expressed in this article are solely those of the authors and do not necessarily represent those of their affiliated organizations, or those of the publisher, the editors and the reviewers. Any product that may be evaluated in this article, or claim that may be made by its manufacturer, is not guaranteed or endorsed by the publisher.
