# Supplementary material for: Aerobic Composting and Anaerobic Digestion Decrease the Copy Numbers of Antibiotic-Resistant Genes and the Levels of Lactose-Degrading Enterobacteriaceae in Dairy Farms in Hokkaido, Japan
Source: Front Microbiol. 2021 Sep 30;12:737420. doi: 10.3389/fmicb.2021.737420 (PMC8515179; doi:10.3389/fmicb.2021.737420)
Supplement: Supplementary file 1 [file Presentation_1.PPTX]

## Slide 1
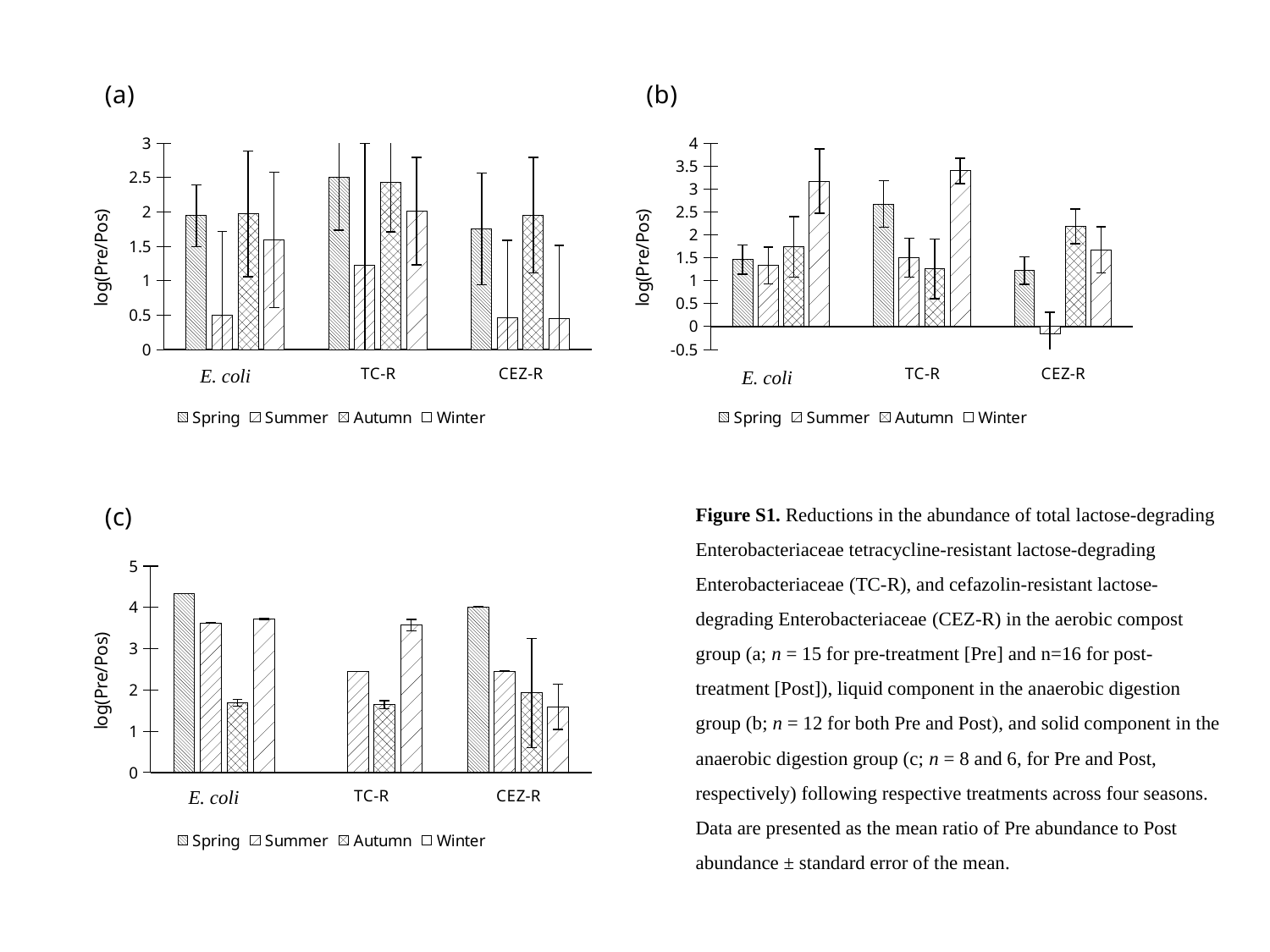

### Chart: (a)
| Category | Spring | Summer | Autumn | Winter |
|---|---|---|---|---|
| E. coli | 1.9452982507218475 | 0.4984182543489253 | 1.9719786728176647 | 1.5933232118422338 |
| TC-R | 2.5082668719805294 | 1.2203418593396234 | 2.435223538334024 | 2.0112281959960763 |
| CEZ-R | 1.753601794194983 | 0.46386426242645484 | 1.9519093925082776 | 0.4464318812210518 |
### Chart: (b)
| Category | Spring | Summer | Autumn | Winter |
|---|---|---|---|---|
| E. coli | 1.4604624447142063 | 1.3312066344540743 | 1.7413820567645442 | 3.171169971849116 |
| TC-R | 2.6712265657637304 | 1.5011788911616701 | 1.2559607551419913 | 3.394834051503189 |
| CEZ-R | 1.2196472816918644 | -0.15960370621634556 | 2.183104043066733 | 1.674216251624058 |E. coli
E. coli
### Chart: (c)
| Category | Spring | Summer | Autumn | Winter |
|---|---|---|---|---|
| E. coli | 4.332821830087832 | 3.6208094273408014 | 1.6895997447016717 | 3.71804154932099 |
| TC-R | 0.0 | 2.449969008676048 | 1.6419826367857726 | 3.5694669701284623 |
| CEZ-R | 4.013788284485633 | 2.4559319556497243 | 1.925224379199179 | 1.5890566261573158 |Figure S1. Reductions in the abundance of total lactose-degrading Enterobacteriaceae tetracycline-resistant lactose-degrading Enterobacteriaceae (TC-R), and cefazolin-resistant lactose-degrading Enterobacteriaceae (CEZ-R) in the aerobic compost group (a; n = 15 for pre-treatment [Pre] and n=16 for post-treatment [Post]), liquid component in the anaerobic digestion group (b; n = 12 for both Pre and Post), and solid component in the anaerobic digestion group (c; n = 8 and 6, for Pre and Post, respectively) following respective treatments across four seasons. Data are presented as the mean ratio of Pre abundance to Post abundance ± standard error of the mean.
E. coli

## Slide 2
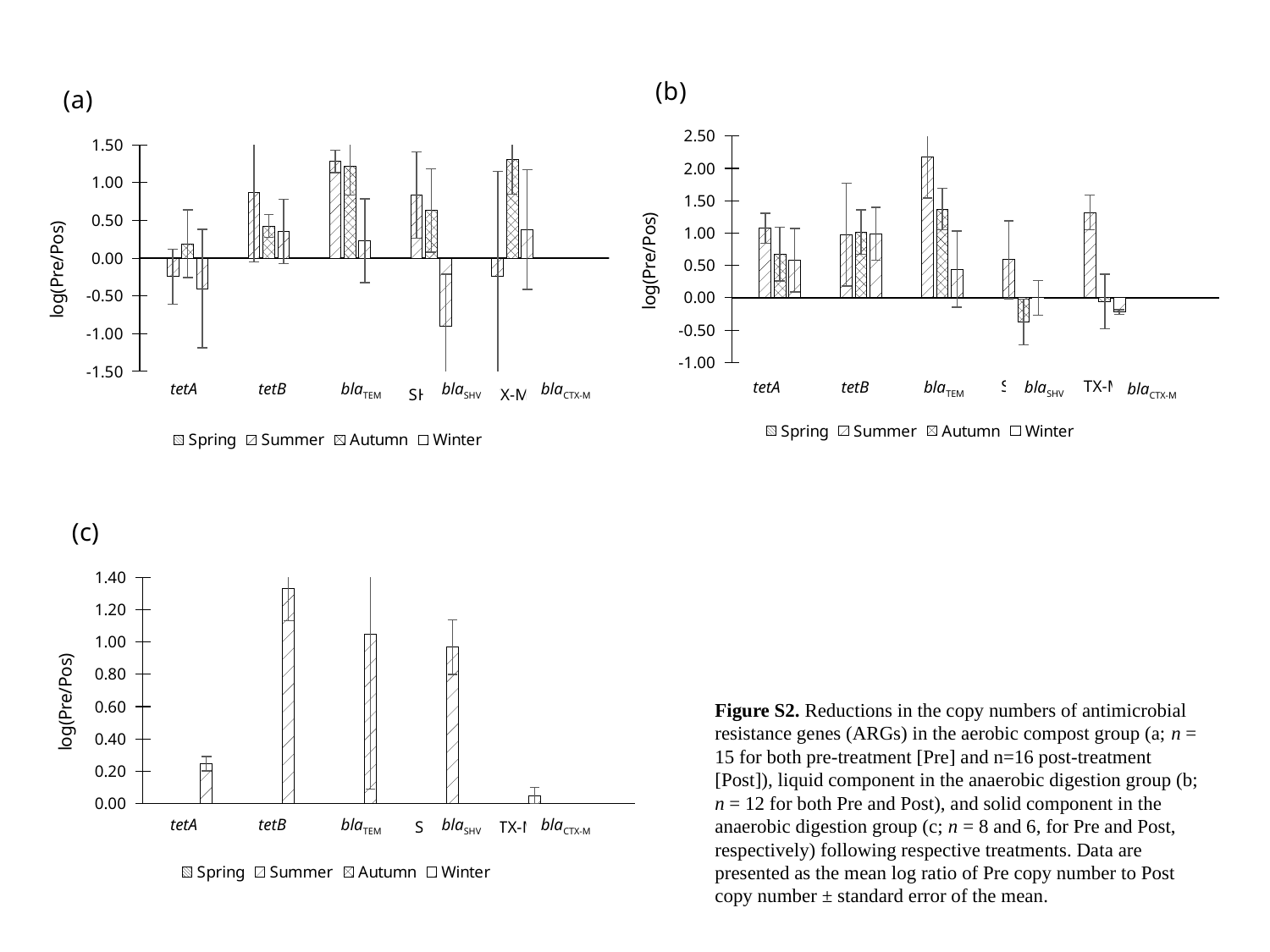

### Chart: (b)
| Category | Spring | Summer | Autumn | Winter |
|---|---|---|---|---|
| tetA | 1.247821151043799 | 1.072820253389225 | 0.6776015636153243 | 0.578637187099616 |
| tetB | 0.6997582525807275 | 0.9763650212700611 | 1.0162147565127446 | 0.9888876152640611 |
| TEM | 1.2623502380686749 | 2.171721949590344 | 1.3707940109443824 | 0.44315857746408466 |
| SHV | 0.21418011969991801 | 0.588800213748295 | -0.37360178392422866 | 0.00015951000166284737 |
| CTX-M | 1.3805207706300469 | 1.3197342801408949 | -0.055438736793138155 | -0.21873301503054687 |
### Chart: (a)
| Category | Spring | Summer | Autumn | Winter |
|---|---|---|---|---|
| tetA | -0.5239556108348853 | -0.24486161073907842 | 0.1907343503601846 | -0.40370942313130853 |
| tetB | 0.06621218525415185 | 0.8655850287091107 | 0.4250896408850606 | 0.35301289995431606 |
| TEM | -0.952411331276529 | 1.2822975605804068 | 1.218613660538714 | 0.23230532618504096 |
| SHV | -0.278762171843395 | 0.8370433072890602 | 0.6320052359175372 | -0.8999215712202279 |
| CTX-M | -0.41679091910005217 | -0.2388370112776208 | 1.304752264323954 | 0.37843276511151336 |tetA
tetB
blaTEM
blaSHV
blaCTX-M
tetA
tetB
blaTEM
blaSHV
blaCTX-M
### Chart: (c)
| Category | Spring | Summer | Autumn | Winter |
|---|---|---|---|---|
| tetA | -0.5206352754781722 | 0.5308172248996934 | -0.8374685991869057 | 0.24671345819829593 |
| tetB | 0.17284865203189215 | 1.9148446728148798 | -0.2036607168756266 | 1.3274602483971054 |
| TEM | 0.3447522029484116 | 2.852353841275028 | 1.2028519608543404 | 1.04602279085185 |
| SHV | -0.5472439294721867 | 0.5831607750296088 | -0.2625187292380191 | 0.9675962334098447 |
| CTX-M | 1.6510344279432445 | 1.5301364944331288 | 0.2854475849011404 | 0.0498711274269652 |Figure S2. Reductions in the copy numbers of antimicrobial resistance genes (ARGs) in the aerobic compost group (a; n = 15 for both pre-treatment [Pre] and n=16 post-treatment [Post]), liquid component in the anaerobic digestion group (b; n = 12 for both Pre and Post), and solid component in the anaerobic digestion group (c; n = 8 and 6, for Pre and Post, respectively) following respective treatments. Data are presented as the mean log ratio of Pre copy number to Post copy number ± standard error of the mean.
tetA
tetB
blaTEM
blaSHV
blaCTX-M
tetA
tetB
blaSHV
16S rRNA gene
blaTEM
blaCTX-M

## Slide 3
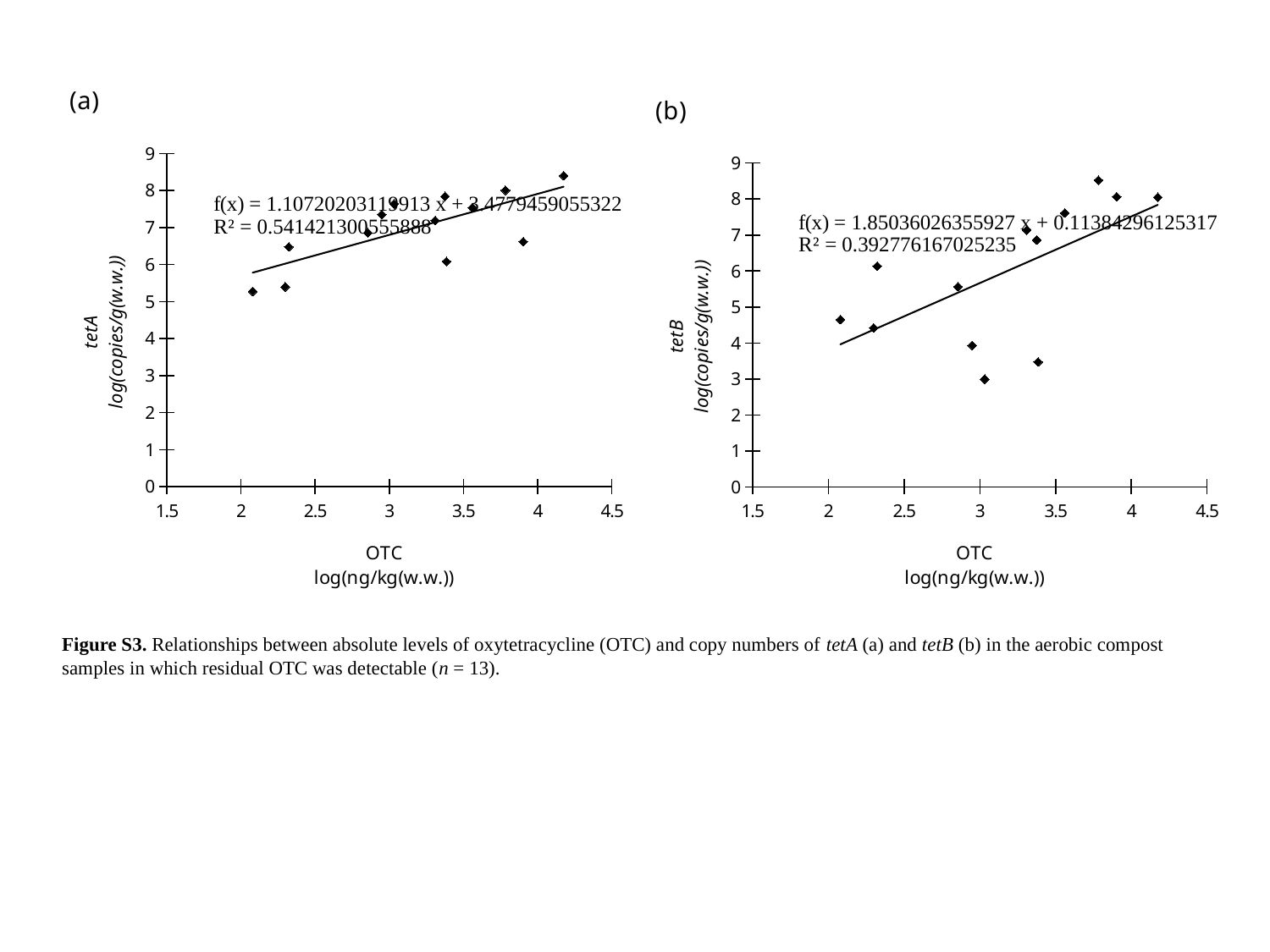

### Chart: (a)
| Category | tetA |
|---|---|
### Chart: (b)
| Category | tetB |
|---|---|Figure S3. Relationships between absolute levels of oxytetracycline (OTC) and copy numbers of tetA (a) and tetB (b) in the aerobic compost samples in which residual OTC was detectable (n = 13).
