# Supplementary material for: Aerobic Composting and Anaerobic Digestion Decrease the Copy Numbers of Antibiotic-Resistant Genes and the Levels of Lactose-Degrading Enterobacteriaceae in Dairy Farms in Hokkaido, Japan
Source: Front Microbiol. 2021 Sep 30;12:737420. doi: 10.3389/fmicb.2021.737420 (PMC8515179; doi:10.3389/fmicb.2021.737420)
Supplement: Supplementary file 2 [file Data_Sheet_1.docx]

**Table S1.** Samples tested in this study

| Farm | Location | Breeding number | Treatment methods | Pre-treatment | Post-treatment |
| --- | --- | --- | --- | --- | --- |
| A | Ebetsu city | 650 | Composting | 4 times | 4 times |
|  |  |  | Anaerobic digestion: liquid | 4 times | 4 times |
|  |  |  | Anaerobic digestion: solid | 4 times | 4 times |
| B | Ebetsu city | 600 | Composting | 3 times* | 4 times |
|  |  |  | Anaerobic digestion: liquid | 4 times | 4 times |
|  |  |  | Anaerobic digestion: solid | 4 times | 2 times* |
| C | Sapporo city | 65 | Composting | 4 times | 4 times |
| D | Ebetsu city | 140 | Composting | 4 times | 4 times |
|  |  |  | Anaerobic digestion: liquid | 4 times | 4 times |
| * One sample subjected to aerobic composting and two solid samples subjected to anaerobic digestion (anaerobic digestion: solid) in farm B were not applicable for analysis. | | | | | |

**Table S2.** Laboratory strains used in this study

| Strain | Bacteria | Origin | Target | Reference |
| --- | --- | --- | --- | --- |
| TC1-2 | *E. coli* | Dairy cattle | *tetA* | 1 |
| T116 | *E. coli* | River water | *tetB* | 2 |
| 133 | *E. coli* | Dairy cattle | *bla_TEM_* | 3 |
| C57 | *K. pneumoniae* | Fly | *bla_SHV_* | 4 |
| 133 | *E. coli* | Dairy cattle | *bla_CTX-M_* | 3 |

1. Sato T, Okubo T, Usui M, Yokota S, Izumiyama S, Tamura Y. (2014). Association of veterinary third-generation cephalosporin use with the risk of emergence of extended-spectrum-cephalosporin resistance in *Escherichia coli* from dairy cattle in Japan. PLOS one. 9, e96101.
2. Tsunoda R, Usui M, Tagaki C, Fukuda A, Boonla C, Anomasiri W, Sukpanyatham N, Akapelwa M L, Nakajima C, Tamura Y, Suzuki Y. (2021). Genetic characterization of coliform bacterial isolates from environmental water in Thailand. J. Infect. Chemother. 27, 722-728.
3. Usui M, Iwasa T, Fukuda A, Sato T, Okubo T, Tamura Y. (2013). The role of flies in spreading the extended-spectrum beta-lactamase gene from cattle. Microb. Drug Res. 19, 415-420.
4. Fukuda A, Usui M, Okubo T, Tagaki C, Sukpanyatham N, Tamura Y. (2018). Co-harboring of cephalosporin (*bla*)/colistin (*mcr)* resistance genes among Enterobacteriaceae from flies in Thailand. FEMS Microbiol. Lett. 365, fny178.

**Table S3.** Primers used in this study

| Target | Purpose | Sequence (5′-3′) | Tm (°C)* | Length (bp) | References |
| --- | --- | --- | --- | --- | --- |
| *tetA* | *tetA* for standard | GGCGGTCTTCTTCATCATGC | 64 | 502 | 1 |
|  |  | CGGCAGGCAGAGCAAGTAGA |  |  |  |
| *tetB* | *tetB* for standard | CAGTGCTGTTGTTGTCATTAA | 55 | 571 | 2 |
|  |  | GCTTGGAATACTGAGTGTTAA |  |  |  |
| *bla_TEM_* | *blaTEM* for standard | ATGAGTATTCAACATTTTCG | 50 | 861 | 3 |
|  |  | TTACCAATGCTTAATCAGTG |  |  |  |
| *bla_SHV_* | *blaSHV* for standard | ATGCGTTATATTCGCCTGTG | 55 | 841 |  |
|  |  | TTAGCGTTGCCAGTGCTCGA |  |  |  |
| *bla_CTX-M_* | *blaCTX-M* for standard | GGTTAAAAAATCACTGCGTC | 50 | 864 | 4 |
|  |  | TTGGTGACGATTTTAGCCGC |  |  |  |
| *tetA* | *tetA* for qPCR | CGGTCTTCTTCATCATGCAAC | 60 | 83 | 1 |
|  |  | GTCCCAGTGAAAGCGATCC |  |  |  |
| *tetB* | *tetB* for qPCR | CAGCAAGTGCGCTTTGGATGCTG | 55 | 101 | 2 |
|  |  | TGAGGTGGTATCGGCAATGA |  |  |  |
| *bla_TEM_* | *blaTEM* for qPCR | GCKGCCAACTTACTTCTGACAACG | 55 | 247 | 3 |
|  |  | CTTTATCCGCCTCCATCCAGTCTA |  |  |  |
| *bla_SHV_* | *blaSHV* for qPCR | CGCTTTCCCATGATGAGCACCTTT | 60 | 110 |  |
|  |  | TCCTGCTGGCGATAGTGGATCTTT |  |  |  |
| *bla_CTX-M_* | *blaCTX-M* for qPCR | ATTCCRGGCGAYCCGCGTGATACC | 62 | 227 | 4 |
|  |  | ACCGCGATATCGTTGGTGGTGCCAT |  |  |  |
| *Tm indicates annealing temperature for PCR/qPCR. | | |  |  |  |
| 1, Lanz R, Kuhnert P, Boerlin P. (2003). Antimicrobial resistance and resistance gene determinants in clinical Escherichia coli from different animal species in Switzerland. Veterinary microbiology. 91(1), 73-84. | | | | | |
| 2, Ma M, Wang H, Yu Y, Zhang D, Liu S. (2007). Detection of antimicrobial resistance genes of pathogenic Salmonella from swine with DNA microarray. Journal of veterinary diagnostic investigation: official publication of the American Association of Veterinary Laboratory Diagnosticians, Inc. 19(2), 161-7. | | | | | |
| 3, Kojima A, Ishii Y, Ishihara K, Esaki H, Asai T, Oda C, et al. (2005). Extended-spectrum-beta-lactamase-producing Escherichia coli strains isolated from farm animals from 1999 to 2002: report from the Japanese Veterinary Antimicrobial Resistance Monitoring Program. Antimicrobial agents and chemotherapy. 49(8), 3533-7. | | | | | |
| 4, Saladin M, Cao VT, Lambert T, Donay JL, Herrmann JL, Ould-Hocine Z, et al. (2002). Diversity of CTX-M beta-lactamases and their promoter regions from Enterobacteriaceae isolated in three Parisian hospitals. FEMS microbiology letters. 209(2), 161-8. | | | | | |

| **Table S4**. qPCR standard curve | | | |
| --- | --- | --- | --- |
|  | Amplification efficiency | R^2^ | Log (limit of quantification) |
| *tetA* | 1.187 | 0.998 | 2.11 |
| *tetB* | 0.905 | 0.997 | 2.84 |
| *bla*_TEM_ | 1.149 | 0.992 | 2.82 |
| *bla*_SHV_ | 0.984 | 0.999 | 2.23 |
| *bla*_CTX-M_ | 1.12 | 0.993 | 2.9 |

| Table S5. Validation of the method characteristics for analyzing antimicrobials in solid samples | | | | |  |
| --- | --- | --- | --- | --- | --- |
| Compound | Recovery (%) (SD, n=3) | LOD (ng/kg) | LOQ (ng/kg) | Calibration range (ng/L) | Correlation coefficient (r^2^) |
| Ampicillin | 77 (13) | 161 | 537 | 0.5–200 | 0.99 |
| Benzylpenicillin | 59 (6) | 50 | 167 | 0.5–200 | 0.99 |
| Cefazolin | 84 (17) | 57 | 190 | 0.5–200 | 0.99 |
| Cefuroxime | 102 (8) | 83 | 276 | 0.5–200 | 0.99 |
| Ceftiofur | 98 (4) | 64 | 214 | 0.5–200 | 0.99 |
| Chlortetracycline | 81 (10) | 32 | 107 | 0.5–200 | 0.99 |
| Doxycycline | 80 (4) | 29 | 98 | 0.5–200 | 0.99 |
| Minocycline | 97 (7) | 29 | 98 | 0.5–200 | 0.99 |
| Oxytetracycline | 63 (6) | 32 | 107 | 0.5–200 | 0.99 |
| Tetracycline | 59 (8) | 38 | 127 | 0.5–200 | 0.99 |

| Table S6. Results of Mantel test in each type of sample | | |
| --- | --- | --- |
|  | *r* | *P* |
| AC | 0.173 | 0.018* |
| Liquid component subjected to AD | 0.293 | 0.004* |
| Solid component subjected to AD | 0.332 | 0.023* |

*r* indicates correlation coefficient between weighted UniFrac distance of bacterial community and Bray-Curtis dissimilarity of 3 dominant ARGs (*tetA*, *tetB*, and *bla*_TEM_). Asterisks (*) indicate significant correlation.
